# Acute Effects of Nordic Hamstring Exercise on Hamstring Stiffness: A Randomised Controlled Trial

**DOI:** 10.3390/jcm14248677

**Published:** 2025-12-07

**Authors:** Gokhan Yagiz, Cristina Monleón, Esedullah Akaras, Sena Adanir, Encarnación Liébana

**Affiliations:** 1 Department of Physiotherapy and Rehabilitation, Faculty of Health Sciences, Amasya University, 05100 Amasya, Turkey; gokhan.yagiz@amasya.edu.tr; 2 Department of Physical Therapy, Faculty of Health Sciences, Tokyo Metropolitan University, Tokyo 92-0397, Japan; 3 Faculty of Physical Education and Sports Sciences, Catholic University of Valencia San Vicente Mártir, 46001 Valencia, Spain; 4 Department of Physiotherapy and Rehabilitation, Faculty of Health Sciences, Erzurum Technical University, 25050 Erzurum, Turkey; 5 Independent Researcher, 25050 Erzurum, Turkey

**Keywords:** injury prevention, lower limb, posterior thigh, sports injuries

## Abstract

**Background**: This study aimed to examine the immediate effects of the Nordic Hamstring Exercise (NHE) on the stiffness of the biceps femoris (BF) and semitendinosus (ST) muscles. **Methods**: This parallel-group randomised controlled trial followed CONSORT 2025 guidelines. Twenty-four physically active adults (16 females, 8 males) were randomly assigned to an NHE group (*n* = 12) or a control group (*n* = 12) using 1:1 gender-stratified randomisation. The NHE group performed 3 sets of 10 repetitions of the NHE, while the control group remained inactive. Muscle stiffness of the BF and ST was assessed pre- and post-intervention using the MyotonPro device. **Results**: No significant overall changes in hamstring stiffness were observed within or between groups (*p* > 0.05). Within the NHE group, the ST tended to increase in stiffness (11.25 N/m, *p* = 0.057), while the BF showed a small, non-significant reduction (−12.00 N/m, *p* = 0.696). The difference in changes between BF and ST was significant (*p* = 0.039). Independent of group allocation, males demonstrated significantly higher baseline stiffness than females for BF (258.13 vs. 195.81 N/m, *p* < 0.001) and for ST (247.88 vs. 174.00 N/m, *p* = 0.003). Regression analysis showed that only height predicted the change in ST stiffness after NHE (R = 0.625, R^2^ = 0.39, *p* = 0.030). **Conclusions**: A single NHE session did not alter overall hamstring stiffness but produced opposite, muscle-specific effects. More research with larger, uniform samples is needed to confirm these findings.

## 1. Introduction

Hamstring strain injuries (HSIs) are widespread and affect athletes of all levels, from amateurs to professionals in sports that require sprinting [1], such as soccer, American football, track and field disciplines, Australian Rules football, and rugby union [2,3,4,5,6]. The HSI rates were estimated as 26% in track and field [7], 24% in soccer [3], 13.7% in Australian football [8], 12% of all American football injuries [4] and 6–15% of all injuries in rugby [9]. Additionally, injury rates have been rising across sports such as soccer, Australian Rules football, and rugby. Additionally, athletes also experience high HSI recurrence rates, such as 32% in American football [10], 16% in soccer [11], 27% in Australian football [12] and 21% in rugby [9]. Importantly, these repeated HSIs are more severe and result in greater time loss than the initial HSIs [9]. In addition to the mentioned reinjury rates, recurrent HSI can cause enthesopathy, issues with the knee menisci, adhesion in the popliteal nerve, motor problems of the ischiatic nerve, abnormal quadriceps strength, and may even end an athletic career [13,14,15]. Therefore, over the past twenty years, scientists have concentrated on understanding the injury mechanism and developing an optimal prevention method for HSIs before their initial occurrences [16,17,18,19,20,21,22,23,24,25].

Most HSIs happen during the late swing phase of high-speed running [26,27,28,29]. The majority of HSIs happen in the biceps femoris muscle (BF), particularly in the long head of the BF, which is part of the hamstrings [30]. It was stated that the hamstrings produce eccentric force to slow down the tibia and control the concentric force of the opposing quadriceps femoris [31]. During the late swing phase of running, the biceps femoris muscle undergoes the greatest elongation among all the hamstring muscles [32]. Additionally, the biceps femoris shows higher muscle activity than the semitendinosus during the late swing phase of running [33]. At this moment, excessive tensile force can cause strains if the muscle fibres cannot withstand the stress [34]. Therefore, scientists have concentrated on enhancing the hamstrings’ limited ability for eccentric contraction and have recommended eccentric strength training as a preventative measure for HSIs [23,24]. Among various eccentric training methods, the Nordic hamstring exercise (NHE) has become increasingly popular [16,18,20].

Medical and sports specialists have been focusing on identifying risk profiles, which involve complex and phenomenal interactions between risk factors, to identify risk patterns of sports injuries [35]. Accordingly, prospective studies have identified a variety of risk factors for HSI, including, but not limited to, injury history (e.g., calf strains [36,37,38,39,40,41], HSI [36,37,41,42,43], knee injuries [44,45], and anterior cruciate ligament injuries [36,37,41], ankle injuries [46], older age [39,42,45,47,48], ethnicity [9,45,48], strength imbalances [10,17,40,47,49,50,51,52,53,54,55,56], fatigue [10,17,57,58,59], lower eccentric strength [44,60,61,62], lower isometric strength [60,61], lower single-leg hamstring bridge (SLHB) performance [44], the lesser single-leg hop distance [61], insufficient warm-up [63], increased anterior pelvic tilt [48,64], altered intermuscular coordination [62,65,66], higher differences between countermovement jumps and non-countermovement jumps [67], increased electromyographic (EMG) activity of the gluteus medius muscle [68], elevated high-speed running exposure [69,70], lesser EMG activity of the trunk muscles [71], thoracic bending at the front-swing phase of sprinting [72], an increment in anterior pelvic tilt at the backswing phase of sprinting [72] and structural risk factors [41,73,74].

Structural risk factors have received attention over the last decade, including muscle architecture, stiffness, and fibre typology [19,41,47,73,74,75]. Muscle architecture has a broad definition in the literature, encompassing the anatomical cross-sectional area, physiological cross-sectional area, fascicle length, muscle thickness, muscle length, and pennation angle (PA) [76]. These skeletal muscle architectural parameters define a muscle’s functional characteristics [77]. Studies have shown that muscle architectural parameters are indicators of strength [78,79,80,81,82,83,84,85,86,87,88,89,90,91], sports performance [92,93,94,95,96,97,98,99,100,101,102,103] and sports injuries [41,75,91,104,105,106,107,108,109,110,111]. Among muscle architectural parameters, shorter biceps femoris fascicle length has been reported as a risk factor for HSIs in soccer players [41]; however, it remains controversial due to its lack of predictive value in American Football players [112]. Regarding the fibre typology, Lievens et al. [73] defined the fast fibre type as a risk factor for HSIs in soccer; on the other hand, Schuermans et al. [113] stated that the fibre typology is not associated with either a history of HSIs or the performance of amateur soccer players. Lastly, increased passive hamstring stiffness has been introduced as one of the factors increasing the risk of HSIs [74]. Stiffness and elasticity of musculoskeletal tissues are fundamental mechanical properties [114]. Muscle stiffness is a crucial factor influencing human movement and sports performance [115]. Muscle stiffness indicates the resistance of the tissue to deformation when force is applied [116]. Normal muscle stiffness maintains a proper balance between flexibility and firmness to enable effective force transfer, improve joint stability, and support movement control, while also holding elasticity to absorb shocks and lower injury risk [117,118,119,120,121].

A portable, phone-sized device called MyotonPro (Myoton AS, Estonia) has recently been introduced to measure the stiffness of superficial musculoskeletal tissues [122,123,124]. This handheld digital device is designed for palpation and includes a feature to measure muscle stiffness [125]. Myotonometry measurements have previously been found to be a valid and reliable tool for assessing muscle stiffness [126,127,128]. The MyotonPro measures by delivering a set of brief mechanical impulses (15 ms, 0.4 N force) while applying a steady pre-compression force of 0.18 N to the tissue layer above the targeted muscle or tendon [129]. Mechanical deformation is applied at the device testing end (d = 3 mm), which is held perpendicular to the skin surface [129]. After a short mechanical impulse, the muscle or tendon responds with a damped oscillation, which is detected by an acceleration sensor connected to the device’s frictionless measurement system [129]. Given its lower cost and portability, the MyotonPRO could serve as an alternative measurement tool for muscle stiffness assessment, which offers a portable and reliable [130] diagnostic and monitoring device in medical practice [131].

The FIFA 11+ warm-up programme, aimed at injury prevention in sports, includes the NHE and recommends using the NHE before training [132]. Therefore, exploring what the immediate effects of the NHE are on the hamstring stiffness via MyotonPro before training, as evaluated in randomised controlled trials, for a higher evidence level [133], may provide new insights into the mechanism of NHE prevention. Considering the information above, this randomised controlled trial aimed to examine the immediate effects of NHE on hamstring stiffness in the physically active population and hypothesised that hamstring muscle stiffness would increase due to the repetitive muscle activity during the NHE.

## 2. Materials and Methods

### 2.1. Design, Timing and Location of Research and Participants

This study was conducted as a parallel group randomised controlled trial with pre-test and post-test measurements for exercise and control groups by following the CONSORT 2025 statement [134]. Participant recruitment and data collection for this study were carried out at the facilities and laboratories of Erzurum Technical University in Türkiye, following approval from the Erzurum Technical University Research and Publication Ethics Committee (Approval number: 2025-ETÜ-0010) on 16 October 2025. Data collection began on the 17th of October and was completed by the 30 October.

Physically active participants were recruited through posters, emails, and verbal advertisements. The inclusion criteria will be considered as (a) being physically active, (b) being aged between 18 and 35, and (c) not having an acute lower extremity injury. There were no gender restrictions for study participants [135]. But randomisation was performed by using a gender stratification via an online randomisation tool feature of GraphPad QuickCalcs (GraphPad Software Inc.; San Diego, CA, USA) [136]. Twenty-four physically active adults (16 females, 8 males) volunteered and met the inclusion criteria. Since more females than males volunteered, the final sample was not balanced in terms of sex in absolute numbers. However, group allocation was performed using gender-stratified randomisation (1:1 for group size, 2:1 for female: male ratio) through the online randomisation tool resulting in equal sex distribution in the NHE (8 females, 4 males) and control (8 females, 4 males) groups. For a 1:1 allocation ratio, either the NHE (intervention) group (*n* = 12, 8 females and 4 males) or the control group (*n* = 12, 8 females and 4 males).

The NHE group performed three sets of 10 repetitions of Nordic Hamstring exercises, with a 3 min break between sets, and the control group did not perform any activities for 15 min between pre-test and post-test. Measurements were taken from the BF and ST muscles just before the first set (pre-test) and immediately after the third set (post-test) for the NHE group. A researcher who did not involve pre-test/post-test stiffness measurements and was not aware of the groups of the participants performed the randomisation to ensure that random allocation concealment was achieved [137]. Participants’ International Physical Activity Questionnaire (IPAQ) short-form scores [138], age, height, weight and dominant side were recorded before the study.

### 2.2. Sample Size

The sample size was determined using the G*Power software (version 3.1.9.7) [139]. A total of 24 participants were deemed sufficient for the study, divided equally into two groups: 12 participants in the intervention group and 12 participants in the control group. This determination was based on statistical analyses using F-tests, ANOVA, repeated-measures analysis, and within-between interactions. assuming a smaller effect size of 0.4 than the previous literature [140], a 0.05 alpha level, 0.95 power, 2 groups with two measurements each, a correlation of 0.5 among measurements, and an epsilon value of 1, which is necessary when analysing fewer than 3 groups.

### 2.3. Hamstring Stiffness Measurements

The MyotonPro device (Version 5.0.0, Serial Number: 1B211097, Myoton AS, Tallinn, Estonia) was used for measuring muscle stiffness in units of N/m. The Myoton Pro device is a digital instrument that features both a body probe and a depth probe with a 3 mm diameter. Myoton Pro begins measurements by applying a gentle initial pressure of 0.18 N to the skin surface via the probe, which then generates a dynamic shear force within the underlying tissue. Following this, the device delivers a brief mechanical pulse of 0.4 N (15 ms) that quickly deforms the tissue [141]. The probe was placed perpendicular to the surface being measured. If any deviations are detected, the device automatically identifies the measurement error and prompts the user to repeat the measurement [141]. The measurement method involves capturing the damped natural vibrations of soft biological tissue via acceleration signals. These signals are then analysed to determine important biomechanical parameters such as muscle tension (T), dynamic stiffness (S), and elasticity (E), which collectively provide insights into the tissue’s stress state and mechanical properties [141].

**Figure 1:**
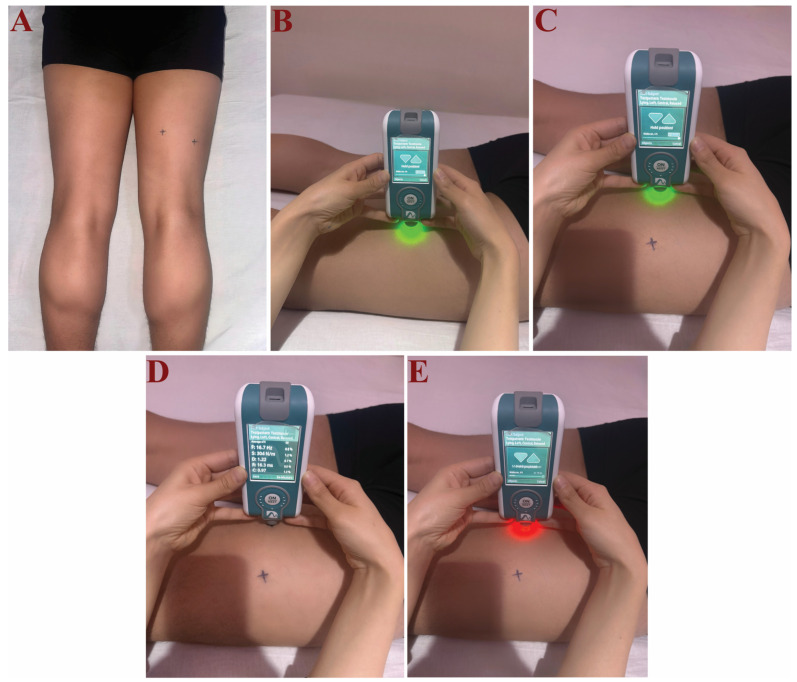
Measurement of muscle stiffness using MyotonPRO. (**A**) measurement points for the semitendinosus at 50% of the distance from the ischial tuberosity to the medial epicondyle of the femur, and for biceps femoris at 50% of the distance from the ischial tuberosity to the medial epicondyle of the femur. (**B**) Measurement for the biceps femoris, where the green light of MyotonPro mentions the correct measurement angle. (**C**) Measurement for the semitendinosus, where the green light of MyotonPro mentions the correct measurement angle. (**D**) The picture shows how the results appear on the device after measurement. (**E**) Image showing an incorrect measurement angle, where the MyotonPro device signals with a red light.

The participants were positioned prone on a standard examination bed, with their hands flat alongside the body and their feet hanging naturally over the edge of the bed to ensure complete relaxation of the hamstring muscles. Stiffness measurements were obtained from the biceps femoris (BF) and semitendinosus (ST) muscles at standardised anatomical locations from the dominant legs [142,143]. The measurement point for the BF will be set at 50% of the distance from the ischial tuberosity to the lateral epicondyle of the femur, and for the ST, at 50% of the distance from the ischial tuberosity to the medial epicondyle of the femur, as illustrated in Figure 1 [143]. The measurement locations were marked with a pencil that remained on the skin until the measurements were finished to ensure that post-test measurements were taken at the same locations as the baseline measurements. Participants were advised not to engage in any physical activities on the day of measurement. They only walked approximately 50 m to the measurement room as a form of physical activity before the assessments, while the control group remained inactive on the bed for the same duration that the NHE group performed NHE. A measurement, consisting of 5 consecutive readings from the Myoton Pro device, was taken at the specified locations by the same researcher and used as the stiffness results for the muscles.

### 2.4. Nordic Hamstring Exercise

**Figure 2:**
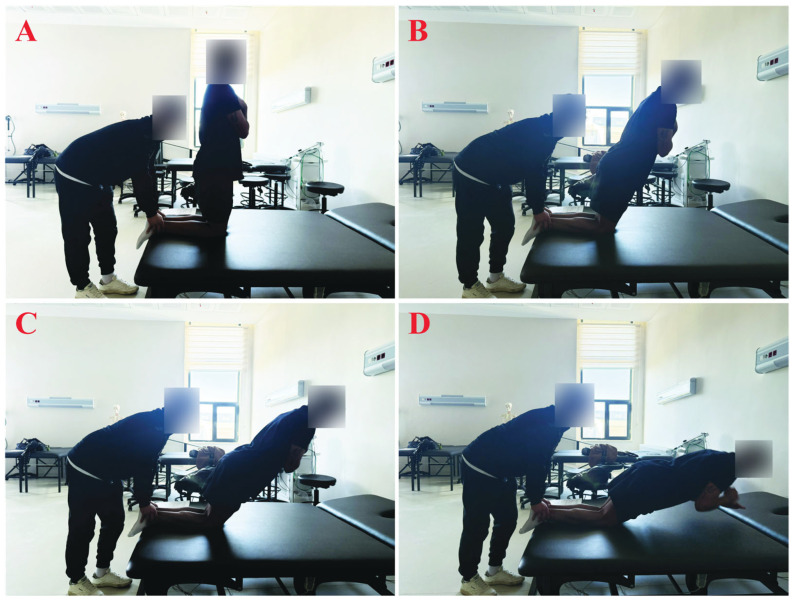
(**A**) Starting position, (**B**) continuum near the initial point, (**C**) continuum close to the final point, and (**D**) near the exercise’s end, where the participant prepares to touch the ground with their hands during the Nordic Hamstring exercise.

Traditional NHE was conducted by the NHE group participants as outlined by Petersen et al. [18]. A partner supported participants’ ankles, applying pressure to ensure their lower legs and ankles stayed in contact with the ground throughout the movement. At the beginning of the movement, when participants’ knees are at 90°, their hips and spines will be in a neutral position (Figure 2). Then, participants initiated a forward-falling motion and eccentrically engaged their muscles to resist this motion. Participants will be expected to slow down their forward-falling movement for as long as possible to reduce their speed of motion. At the end of the movement, participants contacted the floor with their hands and arms, permitting their chest to touch the ground before promptly returning to the starting position by pushing against the floor with their hands and arms to minimise the concentric phase of the movement [144]. To achieve continuous angular velocity, researchers provided instructions to participants as outlined by Burrows et al. [145]. Researchers counted respectively “5 s, 4 s, 3 s, 2 s and 1 s” during the descending movement phase of the NHE from start to finish [145]. The participants will perform 3 sets of 10 NHE repetitions. 3 min breaks will separate sets, and the concentric part of the movement will not be executed [145].

### 2.5. Statistical Analyses

A mixed-model repeated-measures ANOVA was used to compare changes between the intervention and control groups; for each muscle, a one-way ANOVA was employed for descriptive statistics, and linear regression analyses were conducted using IBM SPSS 29.0 software (IBM Corp, Armonk, NY, USA). Furthermore, Hedges’ adjusted *g* effect size was also calculated, which adjusts for potentially biased small sample sizes (*n* < 20), unlike Cohen’s *d* [146]. The Hedges’ *g* effect size was classified as follows: small (0.2), medium (0.5), or large (0.8) [146].

## 3. Results

**Figure 3:**
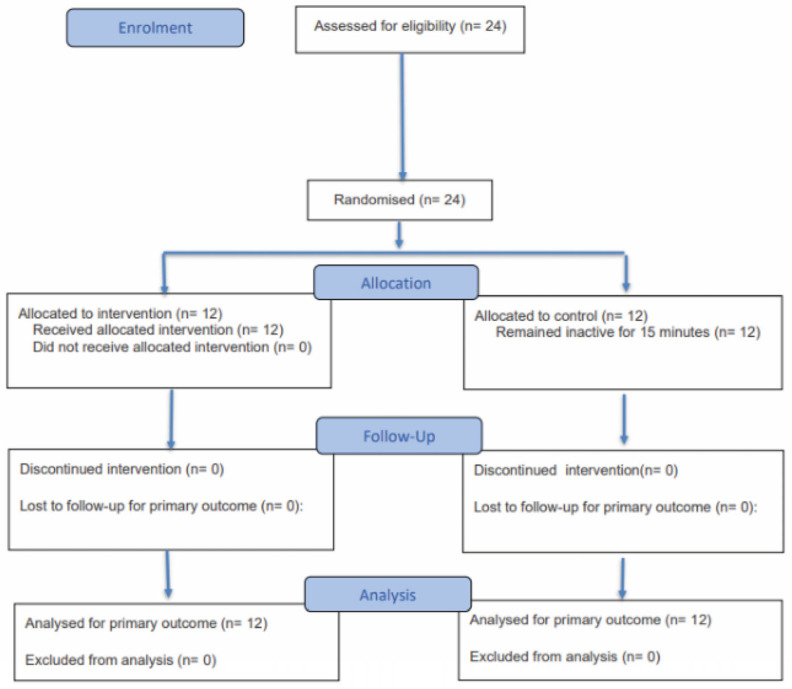
CONSORT 2025 flow diagram.

Twenty-four physically active participants (16 females, 8 males) completed the study without any dropouts or adverse events, as illustrated in Figure 3. IPAQ-sf scores [138] showed that the self-reported physical activity level of each participant was graded as high for each participant, and there were no significant differences detected between groups (*p* = 0.920) (Table 1). Additionally, there were no significant differences in age (*p* = 0.653), body mass (*p* = 0.671), or height (*p* = 0.678) between the groups (Table 1).

As shown in Table 2a, the stiffness of the biceps femoris (BF) slightly decreased in the NHE group (from 213.75 ± 40.27 N/m to 201.75 ± 40.64 N/m), while a small, non-significant increase was seen in the control group (from 219.42 ± 53.75 N/m to 222.25 ± 45.92 N/m). However, neither within-group changes (*p* = 0.696 and *p* = 0.108 for NHE and control, respectively) nor between-group differences (*p* = 0.773–0.157 across parameters) reached statistical significance. The effect sizes ranged from small to moderate (Hedges’ *g* = 0.06–0.48).

For the semitendinosus (ST) muscle (Table 2b), stiffness slightly increased in the NHE group from 188.58 ± 66.97 N/m to 199.83 ± 72.24 N/m, while remaining almost unchanged in the control group (208.67 ± 55.21 N/m to 210.33 ± 55.77 N/m). The change within the NHE group was nearly significant (*p* = 0.057, *g* = 0.16) but did not meet the 0.05 statistical significance threshold. All between-group differences were non-significant (*p* > 0.05). When both muscles were pooled to represent overall hamstring stiffness (Table 2c), no significant changes were detected between or within groups (all *p* > 0.05). Mean stiffness values remained essentially stable for both groups (NHE: 201.17 ± 50.85 → 200.79 ± 53.80 N/m; Control: 214.04 ± 51.91 → 216.29 ± 49.26 N/m).

Importantly, further investigations showed that when the effects of the NHE on the changes in the stiffnesses of the BF and ST were compared in the NHE group (*n* = 12), a significant difference was found between the changes in the BF and ST (11.25 ± 4.70 vs. −12 ± 8.59, respectively, for the BF and ST, *p* = 0.039), while there was no difference between the same parameters for control group (BF change: 2.83 ± 18.51; ST change: 1.67 ± 22.12, *p* = 0.457).

Regression analyses exploring the links between physical characteristics and pre-test stiffness values are summarised in Table 3a–c. These regression analyses were performed across the entire sample (N = 24) to explore associations between anthropometric/physical activity variables and stiffness, independent of group allocation. Among the predictors, height (*p* = 0.015, R = 0.491 for BF; *p* = 0.025, R = 0.456 for overall hamstrings) and gender (*p* < 0.001 for BF and overall hamstrings; *p* = 0.003 for ST) were significant predictors of pre-test stiffness, accounting for 21–42% of the variance. As shown in Table 3d, a multiple regression analysis using gender and height as predictors significantly explained 36–43% of the variability in baseline stiffness across both individual muscles and the entire hamstrings (*p* = 0.003–0.009). Within the NHE group, regression analyses between anthropometric parameters and the magnitude of change in stiffness (Table 3e–g) revealed that only height was significantly associated with changes in ST stiffness (*p* = 0.030, R = 0.625, R^2^ = 0.39). No other relationships between stiffness changes and age, gender, body mass, or physical activity level were significant (*p* > 0.05). However, there was a significant regression between height and gender (R = 0.751, R^2^ = 0.52, *p* = 0.005), which suggests the results should be interpreted cautiously.

Independent of the group allocation, when this study examined pre-test stiffness results of the whole sample based on gender, it found that males (*n* = 8) showed significantly higher muscle stiffness results than females for the BF (258.13 ± 50.61 N/m vs. 195.81 ± 27.30 N/m, *p* < 0.001), ST (247.88 ± 63.90 N/m vs. 174.00 ± 42.88 N/m, *p* = 0.003) and overall hamstring stiffness (253.00 ± 52.36 N/m vs. 184.91 ± 31.79 N/m, *p* < 0.001). Males and females exhibited There were significant differences in height of males and females (179.38 ± 5.80 cm vs. 162.88 ± 6.40 cm, *p* < 0.001). These results may indicate that the significance of height as a predictor of stiffness could be due to gender differences. However, the height of the participants was the only significant predictor of the changes in the stiffness of the ST from baseline (*p* = 0.030, *R* = 0.625, *R*^2^ = 0.39) in the NHE group, which may highlight a necessity for a future study investigating relationships between anthropometric parameters and stiffness in homogeneous populations regarding gender.

## 4. Discussion

This randomised controlled study examined whether 30 repetitions (3 sets of 10) of the Nordic Hamstring Exercise acutely alter hamstring stiffness and whether individual characteristics influence these responses. At the group level, the intervention did not produce a significant overall pre- to post-change in biceps femoris (BF) or semitendinosus (ST) stiffness compared with the control condition, but a notable within-group divergence emerged in the NHE group: ST stiffness tended to increase while BF stiffness tended to decrease (∆ST − ∆BF ≈ +23 N/m, *p* = 0.039). Baseline stiffness was higher in males and taller individuals, and height predicted the extent of ST change. These findings are consistent with the idea that hamstrings are mechanically diverse, with eccentric loading redistributing tension across heads rather than causing a uniform shift in overall hamstring stiffness.

Most recently, Pieters et al. [147] found that eccentric training led to ST stiffness, with a significant incremental trend starting immediately after measurements (+0.20 kPa) and peaking at 48 h (+0.68 kPa) after the exercise, whereas changes were not significant in other hamstring muscles, including BF long head, which had an insignificant slight decrease immediately after the exercise (−0.01 kPa). The finding of Pieters et al. [147] partially aligns with the findings of this study. Similarly, Zhi et al. [148] and Kawakama et al. [149] found that the biceps femoris long head passive muscle stiffness was acutely reduced after eccentric knee flexion when it was assessed with the SWE, which aligns with the findings of this study. Kawakama et al. [149] supported the idea that a plausible physiological explanation for the observed reduction in passive and active stiffness of the biceps femoris long head after eccentric exercise is the temporary remodelling of the intramuscular connective tissue network. Eccentric contractions exert simultaneous longitudinal and transverse strains on the perimysium and endomysium due to muscle elongation and radial expansion during active shortening [150]. This bidirectional deformation fundamentally differs from the uniaxial stretching that occurs during passive lengthening and may temporarily disrupt collagen cross-linking within the mesh-like connective matrix [151,152]. Consequently, the tissue’s ability to resist shear may decline immediately after eccentric loading, which may cause an abrupt decrease in stiffness. The BF, with its greater, thicker and more complex aponeurosis than the semitendinosus, could be particularly susceptible to radial expansion and microstructural laxity. Conversely, the more fusiform-parallel structure of ST, which has a narrower cross-section and less transverse deformation, may maintain or even increase its stiffness under identical conditions, considering the NHE-leading primarily ST activation activity [153,154,155,156]. This connective tissue reaction supports the neuromechanical explanation for the different fatigue responses observed between hamstring heads and offers a structural basis for the temporary softening of the BF documented in this study.

On the other hand, Podczarska-Głowacka et al. [157] reported that six sets of five repetitions (a total of 30 repetitions) resulted in immediate increases in hamstring stiffness due to fatigue accumulation when assessed by MyotonPro. Podczarska-Głowacka et al. [157] reported a significant increase in the BF and ST after the NHE protocol in a one-group repeated measures design. The waiting period between 6 bouts of 5 reps of NHE was approximately 2 min in the study of Podczarska-Głowacka et al. [157] (a total of 10 min of waiting period), while our study employed a 3 min waiting period between sets (a total of 6 min), which could affect the differences. Importantly, Podczarska-Głowacka et al. [157] mentioned significantly biceps femoris long head stiffness compared to semitendinosus, which does not align with the findings of this study, where the ST stiffness tended to increase and BF tended to decrease, with a significant difference between them in the NHE group. This should be further assessed in future studies, accounting for possible anthropometric differences among participants from different countries.

Morphological factors further influenced stiffness responses. Males and taller individuals exhibited higher baseline stiffness, and height was a predictor of the extent of ST adaptation. Longer muscle-tendon units may experience greater strain under identical external loads, thereby increasing passive stiffness following eccentric exposure. Similar effects dependent on morphology have been documented for tendon stiffness and fascicle strain under load, indicating that body size should be considered when prescribing eccentric training volumes. Consequently, standardised repetition-based NHE dosing may result in uneven internal stress distribution among individuals.

From a practical standpoint, the lack of a global stiffness rise and a decremental stiffness trend, thereby a possible improvement in BF compliance, may play a role in the preventive mechanism of NHE and is unlikely to acutely elevate muscle strain risk by decreasing compliance. This finding supports the safety of incorporating NHE within warm-up routines. However, the ST-specific stiffening and BF softening suggest that chronic preventive adaptations attributed to NHE cannot be inferred from a single bout. The literature commonly found no significant alterations in the BF long head or ST after long-term eccentric training programmes [158,159,160].

Several limitations should be acknowledged when interpreting these results. The sample size was small and consisted of physically active, non-elite adults, which limits the external validity to elite or highly trained populations. Additionally, this study could not blind the assessors and participants due to limited field personnel for data collection and the detailed verbal and written explanations provided to participants before enrolment. Measurements were taken solely immediately after exercise, excluding later time points (such as 90 to 120 min or 24, 48 h), when secondary oedema, inflammation, or connective-tissue reorganisation could affect stiffness profiles. A significant methodological limitation is relying solely on MyotonPRO as the mechanical assessment tool. A recent comparative study [161] has shown that MyotonPRO and SWE measure different mechanical properties. Myoton assesses the viscoelastic response of superficial tissues to an external impulse, while SWE measures internal shear-wave propagation within the muscle [161]. Their correlation is weak to moderate and varies across sites, particularly when muscle thickness, pennation angle, or subcutaneous tissue changes. Consequently, Myoton-derived “stiffness” should be viewed as an indicator of combined muscle tone and superficial elasticity, rather than a direct measure of internal muscle stiffness as SWE provides. In thicker muscles, signals from Myoton may be weakened by depth and tissue anisotropy, leading to an underestimation of actual intramuscular stiffness. These methodological differences may partly account for the lack of overall changes observed in the current study

Furthermore, the Myoton device is sensitive to probe pressure, orientation, and relaxation level, making consistent placement over curved posterior thigh surfaces challenging. The lack of simultaneous elastography or electromyography data limits the ability to distinguish structural stiffness from neural tone. Future research should integrate modalities, including Myoton, SWE, EMG, MRE, and muscle architecture measurements, to cross-verify mechanical behaviour and determine whether observed changes are due to functional adaptation or measurement artefacts. The sample consisted of twice as many females as males. While sex was evenly distributed between the NHE and control groups through gender-stratified randomisation, the small male subgroup limited comprehensive sex-specific analyses and reduced the applicability of the results to male athletes.

Finally, variables like differences in hip-knee joint angles, hydration levels, temperature, and previous eccentric exposure were not completely controlled. Despite the previous MyotonPro showing a high to excellent reliability for hamstring stiffness measurements [130], this study did not conduct reliability assessments prior to the study, which is another significant limitation of this study. Therefore, these conclusions should be viewed as specific to short-term, moderate-load NHE in recreational adults assessed by MyotonPro, rather than as conclusive proof of actual intramuscular mechanical change.

## 5. Conclusions

This randomised controlled trial tentatively indicates that a single session of the NHE may produce distinct immediate mechanical responses across hamstring muscles rather than a uniform change in stiffness. Although overall hamstring stiffness did not significantly change immediately after exercise, the semitendinosus tended to increase in stiffness, while the biceps femoris appeared to become more compliant. Although these differences were modest, they reached statistical significance when comparing changes within groups, suggesting that eccentric loading through the NHE might shift mechanical stress towards the medial hamstring, likely because of its dominant activation during the movement. Nevertheless, these findings warrant cautious interpretation. The observed trends may merely indicate transient viscoelastic modifications of superficial tissues rather than authentic intramuscular mechanical remodelling, considering that stiffness was assessed using the MyotonPro device, which predominantly detects superficial and tone-related characteristics. Furthermore, the limited sample size, mixed-gender sample, and brief measurement period constrain the broader applicability of these results.

Collectively, the findings do not endorse the notion of an immediate, uniform stiffening of the hamstrings following NHE; rather, they indicate a complex, muscle-specific mechanical response. This may contribute to a reduced risk of lateral hamstring strain injuries, particularly considering the vulnerability of the biceps femoris long head. Nonetheless, in the absence of long-term follow-up or concurrent electromyographic and elastographic validation, definitive causal inferences remain unwarranted. Future investigations involving larger, more homogeneous cohorts and employing multimodal stiffness evaluation methods (such as the integration of Myoton, SWE, and EMG) are essential to ascertain whether the observed differential stiffness responses are transient mechanical phenomena, early indicators of muscle adaptation, or measurement artefacts.

## Figures and Tables

**Table 1:** Physical characteristics of the participants (mean ± SD).

	Age (Years)	Body-Mass (kg)	Height (cm)	IPAQ MET Score	Gender	Dominant Side
NHE Group	20.00 ± 1.04	61.92 ± 11.35	167.50 ± 10.29	9117 ± 4932	8 females, 4 males	1 left, 11 right
Control Group	19.83 ± 0.72	64.08 ± 13.21	169.25 ± 10.08	8941 ± 3495	8 females, 4 males	12 right
Significance between groups	*p* = 0.653	*p* = 0.671	*p* = 0.678	*p* = 0.920	N/A	N/A

Abbreviations: IPAQ, International Physical Activity Questionnaire; MET, Metabolic Equivalent of Task minutes per week; N/A: Not applicable; NHE, Nordic Hamstring exercise; SD, standard deviation.

**Table 2:** (**a**) Changes in Biceps Femoris muscle stiffness between groups (mean ± SD in units of N/m); (**b**) Changes in Semitendinosus muscle stiffness between groups (mean ± SD in units of N/m); (**c**) Overall changes in hamstring muscle stiffness between groups (mean ± SD in units of N/m).

**(a)**				
	**Pre-Test**	**Post-Test**	**Changes Between Pre-Test and Post-Test**	***p*** **-Value and Effect Sizes for Within-Groups**
NHE Group	213.75 ± 40.27	201.75 ± 40.64	−12.00 ± 29.77	*p* = 0.696, *g* = 0.30
Control Group	219.42 ± 53.75	222.25 ± 45.92	2.83 ± 18.51	*p* = 0.108, *g* = 0.06
*p*-value and effect sizes for between-groups	*p* = 0.773, *g* = 0.30	*p* = 0.259, g = 0.48	*p* = 0.157, *g* = 0.37	N/A
**(b)**				
	**Pre-Test**	**Post-Test**	**Changes Between Pre-Test and Post-Test**	***p*** **-Value and Effect Sizes for Within-Groups**
NHE Group	188.58 ± 66.97	199.83 ± 72.24	11.25 ± 16.29	*p* = 0.057, *g* = 0.16
Control Group	208.67 ± 55.21	210.33 ± 55.77	1.67 ± 22.12	*p* = 0.769, *g* = 0.03
*p*-value and effect sizes for between-groups	*p* = 0.431, *g* = 0.33	*p* = 0.694, *g* = 0.16	*p* = 0.240, *g* = 0.49	N/A
**(c)**				
	**Pre-Test**	**Post-Test**	**Changes Between Pre-Test and Post-Test**	***p*** **-Value and Effect Sizes for Within-Groups**
NHE Group	201.17 ± 50.85	200.79 ± 53.80	−0.38 ± 16.79	*p* = 0.934, *g* = 0.01
Control Group	214.04 ± 51.91	216.29 ± 49.26	2.25 ± 14.17	*p* = 0.621, *g* = 0.04
*p*-value and effect sizes for between-groups	*p* = 0.546, *g* = 0.25	*p* = 0.469, *g* = 0.30	*p* = 0.683, *g* = 0.17	N/A

Abbreviations: N/A: Not applicable; N/m, Newtons per metre; NHE, Nordic Hamstring exercise; SD, standard deviation.

**Table 3:** (**a**) Regression between the physical characteristics of the whole sample and pre-test biceps femoris muscle stiffness; (**b**) Regression between the physical characteristics of the whole sample and pre-test semitendinosus muscle stiffness; (**c**) Regression between the physical characteristics of the whole sample and pre-test overall hamstring muscle stiffness; (**d**) Multiple Linear Regression of the significant predictors (gender and height) with pre-test biceps femoris, semitendinosus, and overall hamstring muscle stiffness; (**e**) Regression between the physical characteristics of the intervention (NHE) group and changes in the biceps femoris muscle stiffness; (**f**) Regression between the physical characteristics of the intervention (NHE) group and changes in the semitendinosus muscle stiffness; (**g**) Regression between the physical characteristics of the intervention (NHE) group and changes in the overall hamstring muscle stiffness.

**(a)**		
	***p*** **and *R*-Values**	***R*^2^** **Values**
Age	*p* = 0.664, *R* = 0.094	*R*^2^ = 0.01
Body-mass	*p* = 0.233, *R* = 0.253	*R*^2^ = 0.06
Height	*p* = 0.015 *, *R* = 0.491	*R*^2^ = 0.24
Gender	*p* < 0.001 **, *R* = 0.645	*R*^2^ = 0.42
Physical activity level (IPAQ MET score)	*p* = 0.456, *R* = 0.160	*R*^2^ = 0.03
**(b)**		
	***p*** **and *R*-Values**	***R^2^*** **Values**
Age	*p* = 0.728, *R* = 0.075	*R*^2^ = 0.01
Body-mass	*p* = 0.301, *R* = 0.220	*R*^2^ = 0.05
Height	*p* = 0.064, *R* = 0.385	*R*^2^ = 0.15
Gender	*p* = 0.003 *, *R* = 0.584	*R*^2^ = 0.34
Physical activity level (IPAQ MET score)	*p* = 0.298, *R* = 0.222	*R*^2^ = 0.05
**(c)**		
	***p*** **and *R*-Values**	***R*^2^** **Values**
Age	*p* = 0.683, *R* = 0.088	*R*^2^ = 0.01
Body-mass	*p* = 0.242, *R* = 0.248	*R*^2^ = 0.06
Height	*p* = 0.025 *, *R* = 0.456	*R*^2^ = 0.21
Gender	*p* < 0.001 **, *R* = 0.419	*R*^2^ = 0.39
Physical activity level (IPAQ MET score)	*p* = 0.333, *R* = 0.207	*R*^2^ = 0.04
**(d)**		
Gender and height for pre-test BF stiffness	*p* = 0.003 *, *R* = 0.646	*R*^2^ = 0.42
Gender and height for pre-test ST stiffness	*p* = 0.009 *, *R* = 0.599	*R*^2^ = 0.36
Gender and height for pre-test overall hamstring stiffness	*p* = 0.003 *, *R* = 0.654	*R*^2^ = 0.43
**(e)**		
	***p*** **and *R*-Values**	***R*^2^** **Values**
Age	*p* = 0.170, *R* = 0.424	*R*^2^ = 0.18
Body-mass	*p* = 0.939, *R* = 0.025	*R*^2^ = 0.001
Height	*p* = 0.597, *R* = 0.170	*R*^2^ = 0.03
Gender	*p* = 0.630, *R* = 0.155	*R*^2^ = 0.02
Physical activity level (IPAQ MET score)	*p* = 0.674, *R* = 0.136	*R*^2^ = 0.02
**(f)**		
	***p*** **and *R*-Values**	***R^2^*** **Values**
Age	*p* = 0.655, *R* = 0.144	*R*^2^ = 0.02
Body-mass	*p* = 0.277, *R* = 0.341	*R*^2^ = 0.12
Height	*p* = 0.030 *, *R* = 0.625	*R*^2^ = 0.39
Gender	*p* = 0.333, *R* = 0.306	*R*^2^ = 0.09
Physical activity level (IPAQ MET score)	*p* = 0.632, *R* = 0.154	*R*^2^ = 0.02
**(g)**		
	***p*** **and *R*-Values**	***R^2^*** **Values**
Age	*p* = 0.334, *R* = 0.306	*R*^2^ = 0.01
Body-mass	*p* = 0656., *R* = 0.144	*R*^2^ = 0.02
Height	*p* = 0.138, *R* = 0.454	*R*^2^ = 0.21
Gender	*p* = 0.368, *R* = 0.286	*R*^2^ = 0.01
Physical activity level (IPAQ MET score)	*p* = 0.543, *R* = 0.195	*R*^2^ = 0.04

Abbreviations: BF, biceps femoris; MET, Metabolic Equivalent of Task minutes; NHE, Nordic Hamstring exercise; ST, semitendinosus; *, statistically significant (*p* < 0.05); **, statistically significant (*p* < 0.001).

## Data Availability

The original contributions presented in this study are included in the article. Further inquiries can be directed to the corresponding author.

## Abbreviations

The following abbreviations are used in this manuscript:

BF	Biceps femoris
HSI	Hamstring strain injuries
NHE	Nordic hamstring exercise
ST	Semitendinosus
